# Effects of Hyrax Maxillary Expander on Nasal Cavity and Upper Airway in Adolescents: A Systematic Review and Meta-Analysis

**DOI:** 10.3390/healthcare12212148

**Published:** 2024-10-29

**Authors:** Zihe Zhao, Rongkai Cao, Mengze Yao, Chongshan Liao

**Affiliations:** Shanghai Engineering Research Center of Tooth Restoration and Regeneration, Tongji Research Institute of Stomatology, Stomatological Hospital and Dental School, Tongji University, Shanghai 200072, China; 2151040@tongji.edu.cn (Z.Z.); caorongkai1998@163.com (R.C.); 2151841@tongji.edu.cn (M.Y.)

**Keywords:** hyrax expander, rapid maxillary expansion, upper airway, nasal cavity

## Abstract

Background: Whether Hyrax maxillary expander is an effective treatment for maxillary transverse deficiency as well as expansion of the upper airway is still controversial. The study’s purpose was to evaluate 3D changes in upper airway dimensions of adolescent patients measured primarily by cone-beam computed tomography (CBCT) after rapid maxillary expansion (RME) with the Hyrax maxillary expander. Methods: Studies up to 1 April 2024 were searched in the following databases: PubMed/MEDICINE, Web of Science, Cochrane Library, and Embase. Inclusion criteria were clinical trials and cohort studies that assessed the effect of RME in adolescent patients with upper airway stenosis using CBCT-based three-dimensional analysis. The risk of bias in the study outcomes was assessed using the Cochrane Collaboration’s risk of bias tool, the GRADE method, and a tool for evaluating non-randomized controlled study (non-RCT) literature from a previous systematic review, depending on the types of articles. The study was reported in accordance with PRISMA guidelines. Results: The study conducted a random effects meta-analysis of mean differences and 95% confidence intervals for changes in upper airway volumes, including the nasal cavity (NC), in the outcomes of 16 included studies, followed by subgroup analyses. Conclusion: A significant increase in nasopharynx volume was observed after rapid maxillary expansion (RME) by Hyrax maxillary expander (MD = 0.69, 95% CI (0.09~1.28), *p* = 0.02).

## 1. Introduction

Maxillary transverse deficiency (MTD) is a common clinical malocclusion; its clinical manifestations are narrow maxillary dental arch, high arch of the palatal lid, unilateral or bilateral posterior crossbite, and crowding of teeth [1]. Adolescent patients with MTD have wider buccal corridors, which affects their aesthetics. If the anterior and posterior crossbites are not corrected during the growth spurt, it can lead to a narrowing of the bony maxillary arch, which is often accompanied by abnormal sagittal development of the maxilla, affecting the patient’s facial shape [2]. Moreover, the narrowed maxilla is often accompanied by a narrow nasal airway, which can easily lead to oral breathing habits. In severe cases, it may cause obstructive sleep apnea syndrome (OSAS), affecting the patient’s oral psychological state and normal life [3]. Among the multiple factors, myofunctional disorders associated with negative oral habits are the most prevalent factors that cause MTD [4]. Depending on the severity, MTD is subdivided into bony disorder and cartilaginous disorder [1].

Maxillary expansion (ME) is an effective method of treating MTD, which involves expanding the mid-palatal suture using orthodontic or surgical methods to widen the width of the maxilla and the dental arch, improve the maxillary palatal width, and match the width of the maxilla and mandible to open gaps for subsequent orthodontic treatment [5]. Rapid maxillary expansion (RME) is a crucial and the most used therapeutic tool in ME treatment [6]. At present, the common RME devices include the Hyrax expansion device, Hass expansion device, Schwarz expansion device, implant-assisted rapid maxillary expansion device, and so on. In previous studies, RME has also been frequently used in conjunction with other treatment modalities to form a combined treatment to ease the symptoms of MTD, like surgically assisted rapid palatal expansion (SARPE) before RME treatments [7]. Retainers and facial masks are also employed after RME to maintain long-term efforts [8]. These techniques are increasingly utilized in adolescent and adult patients past their growth spurts, offering clear outcomes and good stability. However, detailed research or systematic review of the different types of palatal expanders or the effect of one exact type of palatal expander is limited [9].

The Hyrax expander consists of support arms and band rings. The four support arms are welded to the band rings, and the four band rings are bonded to the supporting teeth (usually the maxillary bilateral first bicuspids and first molars), and the spreader is placed sagittally between the first bicuspids and the first molars at 2 mm from the palatal mucosa [10]. A modified version is also available by replacing the band rings on the first premolar with micro-implants in the anterior region [11]. While several papers have compared the effects of Hyrax expanders with other RME expanders on the upper airway, there is no specialized literature that focuses exclusively on the impact of Hyrax expanders on the upper airway [12].

Computed tomography, especially cone beam computed tomography (CBCT), has been one of the most common 3D measuring ways to access the upper airway situation, with considerable advantages in measuring a 3D volume over traditional 2D imaging methods [13]. CBCT offers superior visualization and reliable analysis of upper airway dimensions [14]. Given this, the meta-analysis primarily evaluates research that utilizes CBCT to examine airway changes after the use of a Hyrax expander [15,16,17,18,19,20,21,22,23,24,25,26,27,28,29,30].

RME has been widely used in adolescent patients with MTD, and the efficacy has been significant, especially studies in recent years have more precisely pinpointed some of the changes caused by RME in the upper airway of patients, but there is still no consensus on the effect of RME to the volume and sagittal diameter of the patient’s upper airway and, in particular, on the exact extent of the effect. While some studies have demonstrated improvement in upper airway volume and sagittal diameter with RME [15,16,17,18,19,20,21,22,23,24,25,26,27,28,29], others have found no significant change [30]. Several systematic reviews and meta-analyses have assessed the effects of palatal dilatation on upper airway function and dimensions [12], but none have investigated the effects of the Hyrax expander separately. The method of examining the dilatation is also relatively homogenous. Considering this introduction, the purpose of this review was to examine the changes that occur in the upper airway of adolescent patients after RME treatment with Hyrax expander by evaluating CBCT as well as other measurements from previous literature and to draw out the strengths and weaknesses of Hyrax expander in the maxillary expansion treatment process.

## 2. Materials and Methods

### 2.1. Eligibility Criteria

Registered with PROSPERO under the number CRD42024548370, this study strictly followed the PICOS model of evidence-based medicine, i.e., P (Patients), I (Interventions), C (Comparison), O (Outcomes), and S (Study), is based on the structured decomposition of clinical questions in evidence-based medicine, which is characterized by the sorting out and summarization of dispersed clinical questions, and the purposeful collection of questions [31]. In this review, the inclusion and exclusion criteria were specified based on the following: explicit RME with Hyrax expander (Intervention), definite effect on upper airway morphology and function (Outcomes), and patients in the growth period (Population). Clinical (Study) was included regardless of randomization. Non-Hyrax expander treatment modalities or healthy controls were included as comparison (Comparison). The null hypothesis (H0) of study is that Hyrax expander is an effective treatment to promote the upper airway volume significantly. The study strictly followed PRISMA guidelines during meta-analysis [32].

### 2.2. Literature Search and Selection

We searched four electronic databases (PubMed, Web of Science—WoS, Cochrane Library, Embase) from earliest searchable date to 1 April 2024, using MeSH terms as per the database rules. The search formula for each database is detailed in Table 1. To prevent omissions, fuzzy searches were used on all capable websites. Additionally, since some literature did not specify the bow expander used in the abstract, we also examined all previous systematic reviews to ensure no articles were missed.

Two investigators, Z.Z. and C.L., independently conducted and verified the screening of online literature and data extraction. Any disagreements were discussed and resolved or referred to a third investigator for adjudication. Because researchers have qualified the database in a way that adequately limits country, language, and type of study, the search did not set any other restrictions. The results of healthy individuals or other treatment recipients who served as a control group for the Hyrax expander were included in the literature characteristics as comparisons. After integrating search results from all databases and removing duplicates, we screened the title and abstract to select articles. In cases of the different judgments on inclusion criteria, full-text reading was performed for final assessment to decide whether to include the literature.

### 2.3. Data Extraction

We used Excel 2021 (Microsoft Corp., Redmond, WA, USA) to extract the key data from literature. The data extraction included information on authors, year of publication, language, country, study design, random methods (RCT study only), control group and its methods, treatment group and its methods, retention period, ethical approval, eligibility criteria of patients, measurements and results, nasal cavity borders, conclusion. No inclusion restrictions were set for studies in the program as long as they adhered to the search formula and application of Hyrax expander for the treatment. For studies with incomplete data, authors were contacted via e-mail to obtain the complete information. We conducted a citation search according to the inclusion criteria and checked and equally included the citation literature.

In measurements and results, the usual measurements of the nasal cavity and the upper airway contain measurements of nasopharynx, oropharynx, and hypopharynx three sagittal images anatomically, and they were included in the meta-analysis as three-dimensional airway metrics measured by CBCT. In addition, since much of the literature incorporates airway-related cephalometric markers for two-dimensional studies, these metrics were also included in the study for the sake of completeness and comprehensiveness of the systematic review. The study performed a random effects meta-analysis of mean differences and 95% confidence intervals for changes in upper airway volume, including nasal cavity (NC) of the included study outcomes in the meta-analysis, followed by subgroup analyses.

### 2.4. Risk of Bias of Included Studies

We utilized the Cochrane Risk of Bias Assessment Tool to adjust the risk of bias of included RCT studies. Each entry was individually rated as “low risk”, “uncertain” and “high risk”. The preexisting meta-analysis literature tailors the tool developed specifically for systematic reviews to customize the risk of bias of the included non-randomized trials based on empirical evidence that includes a variety of assessment tools for the Newcastle-Ottawa Scale and orthodontic clinical bias [33,34,35,36]. Due to the large number of non-randomized controlled trials (non-RCTs) included in this article, this meta-analysis opted to use this tool to evaluate the inclusion of non-RCTs in the same way.

### 2.5. Method of Meta-Analysis

In this study, continuous variables were selected using standardized mean difference (*SD*) and its 95% CI as their statistic, and dichotomous variables were analyzed using relative risk (RR) and its 95% CI as their statistic for outcome analysis. Based on the values we collected, the *SD* (standardized mean difference) for each study can be calculated as follows:

$$SD=\frac{{\bar{x}}_{\exp.}-{\bar{x}}_{\mathrm{control}}}{{SD}_{\mathrm{pooled}}}$$

*SD* value is calculated by dividing the difference between means by the pooled standard deviation (*SD_pooled_*) that can be calculated as below:

$$SD_{\mathrm{pooled}}=\sqrt{\frac{\left(n_{G1}-1\right)s_{G1}^{2}+\left(n_{G2}-1\right)s_{G2}^{2}}{\left(n_{G1}-1\right)+\left(n_{G2}-1\right)}}$$

$n_{G1}$ and $n_{G2}$ are sample sizes of control and treatment groups and $s_{G1}^{2}$ and $s_{G2}^{2}$ are variances [37].

Heterogeneity of studies was assessed using I2 statistics test, and if heterogeneity was high, subgroup analysis and sensitivity analysis were used to assess the reasons. Forest plots and funnel plots were created using Review Manager 5.3 software (The Cochrane Collaboration, London, UK). Statistical analysis was performed using SPSS 26 software (IBM Corp., Armonk, NY, USA). Differences were considered statistically significant when *p* ≤ 0.05.

### 2.6. Methodological Quality of Included Reviews

The evidence for outcome indicators in the included reviews was evaluated using the GRADEprofiler 3.6 software (Gradepro Corp., Hamilton, ON, Canada). The ratings considered study bias, consistency, indirectness, precision, and other biases. The GRADE quality of evidence was categorized as “high”, “intermediate”, “low” and “very low”. The RCT studies per se were of high-quality evidence and thus were downgraded individually according to these ratings.

## 3. Results

### 3.1. Search Results

**Figure 1:**
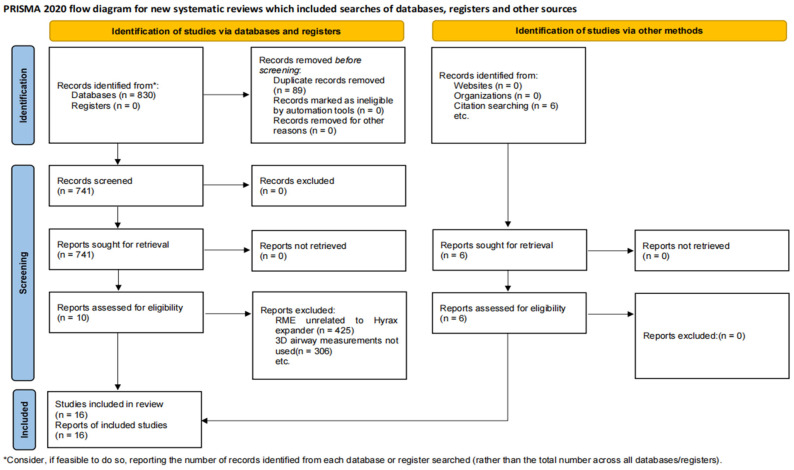
Flow chart of the literature search and results. From Page et al. [32].

We strictly followed the searching strategy above and obtained a total of 836 papers (the searched databases and the detected number of articles are: PubMed 79 results, Web of Science 208 results, Embase 133 results, Cochrane Library 410 results, Citation searching 6 results). After the following exclusion of 89 papers for duplicates, 712 papers after the title and abstract reading, 35 studies after full-text reading (425 studies unrelated to the Hyrax bow expander, 306 studies without use of three-dimensional imaging means, including CBCT). Sixteen papers were finally included in this systematic review (Figure 1) [38]. Some literature on experiments using the Hyrax expander did not specify the type of the expander in the title or abstract, and in some cases, the Hyrax expander was just one experimental group. To obtain comprehensive data on Hyrax expansion, we used fuzzy searches to broaden the scope of literature screening. Most of the excluded literature involved rapid maxillary expansion unrelated to the Hyrax expander.

### 3.2. Characteristics of Included Studies

The extraction of the features of the literature is specified in Table 2, among which 15 research papers were in English and 1 in Chinese, which the researchers read through native language skill and translation software. The included literature did not detail the ethnic background of the study participants. We listed countries of included studies and infer this information from their countries. Regarding study design, 2 of the 16 papers were RCTs with clear randomization methods, while the remaining 14 were clinical cohort studies, including 8 prospective and 6 retrospective studies.

Among the included studies, 15 had ethical approval, while 1 did not mention [26]. Regarding eligibility criteria, one study did not specify inclusion criteria [17], and six studies listed exclusion criteria [16,21,22,23,25,27]. The 3D imaging measurements were obtained using CBCT images and Spiral CT in the studies. The scanning settings supplied by the studies included scanning time, field of view (FOV), mA, voxel size, kVp, and other information. The software packages used for 3D reconstruction included Dolphin, ITK-SNAP, OsiriX MD, Invivo5, Ez3D2009, and so on.

Table 3 summarizes the outcome metrics; three papers measured cross-sectional area, which is the most used 2D metric for measuring airway changes [18,23,27]. The 3D indicators of upper airway changes measured in the studies included nasal cavity, nasopharyngeal volume, oropharyngeal volume, and hypopharyngeal volume. Additionally, the upper airway measurement sections that are not common to certain individual articles have also been listed in the table.

### 3.3. Risk of Bias of Included Studies

We used the Risk of Cochrane Bias Tools to assess the quality of the two included RCTs. The remaining 14 articles, all of which were cohort study articles, had a higher risk of bias than RCT articles and were assessed using the customization tool mentioned above. None of the studies reported the use of blinding of investigators and measurements, and most outcomes were reported as unclear. The overall quality of the included studies is low. The risk of bias in results is shown in Table 4 and Table 5.

### 3.4. Methodological Quality among Included Studies

Nasal cavity score indicators were graded as moderate, and the remaining indicators were graded as low. It is important to note that this systematic review has a high risk of bias due to the wide distribution of irrelevant variables such as age, country, duration of operation, and specific treatment course. The results of the grading characteristics of the quality of evidence are shown in Table 6. The reasons causing the low quality of evidence were unclear random allocation and blinding in terms of risk of bias and large heterogeneity of nasopharynx in terms of inconsistency (I^2^ > 50%).

### 3.5. Result of Meta-Analysis

In this article, forest plots are used to present the statistically summarized results of the meta-analysis for each metric. In the following forest plots, small squares are estimates of the MD value (i.e., in this article, the change in effector metric volume by bow expansion treatment) for each study included, and the size of the squares indicates the weights assigned. The horizontal line where the small square is located indicates the 95% confidence interval of the OR value; when the horizontal line is to the right of the null line, it indicates that the study factor is positively related to the occurrence of the outcome event (i.e., in this article, the expansion of the effect indicator volume by the Hyrax expander); when the horizontal line is to the left of the null line, it indicates that the study factor is negatively related to the occurrence of the outcome event (i.e., in this article, the failure of the expansion of the effect indicator volume by the Hyrax expander). The diamond indicates the 95% confidence interval for the combined effect value, which is the combined value for the outcome of all included studies. The midpoint of the diamond indicates the point estimate of the combined effect value, and the length indicates the confidence interval for the combined effect value. The I^2^ test was used to measure the proportion of inconsistencies in the combined estimate due to between-study heterogeneity. An I^2^ value of less than 30% represents low heterogeneity, 30–60% moderate heterogeneity, and more than 60% high heterogeneity. The I^2^ value was used to measure the proportion of heterogeneity in the combined estimate due to between-study heterogeneity.

#### 3.5.1. Nasal Cavity

**Figure 2:**
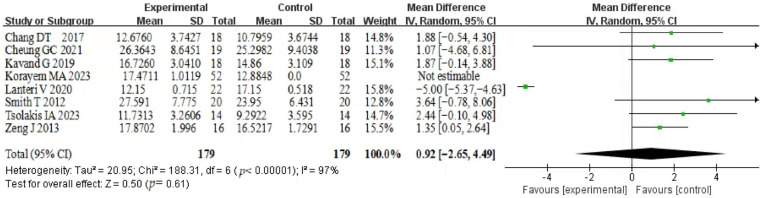
Forest plot of nasal cavity [19,21,22,24,25,28,29,30].

The analysis of data from the nasal cavity showed significant heterogeneity (*p* < 0.00001, I^2^ = 97%), therefore, a random effects model was used. In total, 179 patients from eight studies were included. Nasal cavity showed no significant expansion in volume after the holding period (MD = 1.92, 95% CI (1.06~2.79), *p*= 0.61). The included studies were weighted similarly due to similar numbers, with a range between 11.3% and 15.8%. Only the study of Lanteri et al. showed no significant difference in the variation of nasal cavities [25]. Korayem’s study did not give a clear SD, so his data were not taken to estimate [24]. The 95% confidence intervals for the effect sizes of the studies by Cheung GC and Smith T are outside the range shown in the graphic and are indicated by arrows [21,28]. The results are shown in Figure 2. 

#### 3.5.2. Nasopharyngeal Volume

**Figure 3:**
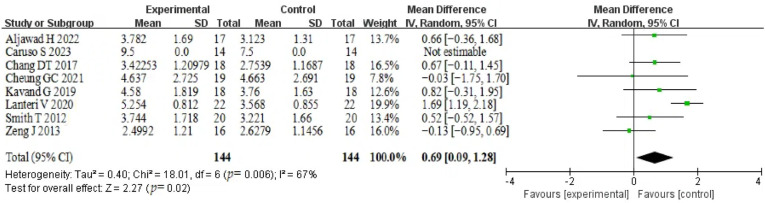
Forest plot of nasopharyngeal volume [16,18,19,21,22,25,28,30].

The analysis of nasopharyngeal volume demonstrated significant heterogeneity (*p* = 0.006, I^2^ = 67%), therefore, a random effects model was used. Finally, 144 patients from eight studies were included. Nasopharyngeal showed significant expansion in volume after the holding period, and the difference was statistically significant (MD = 0.69, 95% CI (0.09~1.28), *p* = 0.02). The included studies were weighted differently due to similar numbers, with a range between 7.8% and 19.9%. Caruso did not give a clear SD, so his data were not taken to estimate [18]. The results are shown in Figure 3.

#### 3.5.3. Oropharyngeal Volume

**Figure 4:**
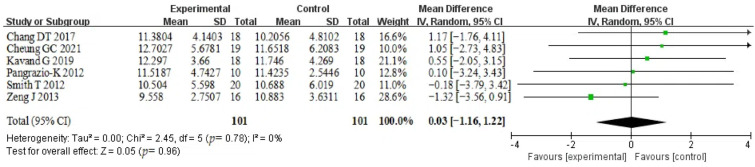
Forest plot of oropharyngeal volume [19,21,22,27,28,30].

Six studies reported differences in oropharyngeal volume before and after treatment in 101 patients, with no significant heterogeneity between groups (*p* = 0.78, I^2^ = 0%); therefore, a fixed-effects model was used. The Hyrax arch expander was able to increase the treatment volume, though not significant, and the difference was not statistically significant when comparing between the two groups (MD = 0.03, 95% CI [−1.16~1.22], *p* = 0.96). Depending on the number of participants in the experiments, the weights assigned to each experiment ranged from 10. 0% to 28.6%, with Zeng’s experiment accounting for the largest weight. Smith T and Zeng J showed different results from the other four studies [28,30]. The 95% confidence intervals for the effect sizes are outside the range shown in the graphic and are indicated by arrows. The results are shown in Figure 4.

#### 3.5.4. Hypopharyngeal Volume

**Figure 5:**
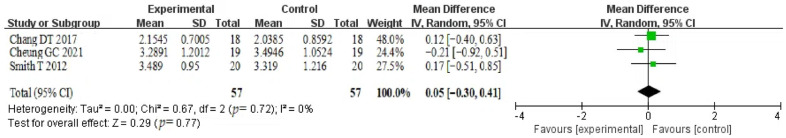
Forest plot of hypopharyngeal volume [19,21,28].

Only three studies reported differences in hypopharyngeal volume before and after treatment in 57 patients without significant heterogeneity between groups (*p* = 0.72, I^2^ = 0%). Therefore, a fixed-effects model was used. The Hyrax arch expander was able to increase the treatment volume, though lacking significance, and the difference was not statistically significant when comparing between the two groups (MD = 0.05, 95% CI [−0.30~0.41], *p* = 0.77). Because of the small sample size included in the analysis, further research on hypopharyngeal is needed. Three studies were weighed with a range between 24.4% and 48.0%. The results are shown in Figure 5.

## 4. Discussion

### 4.1. General Information

The purpose of this article is to provide data on the effects of RME using a Hyrax expander of the upper airway in adolescents. In order to expand the sample size of the data and produce statistically significant findings, we utilized a wide range of inclusion criteria when conducting the search without setting limitations in terms of year of publication or language. The study demonstrated that the Hyrax expander had a significant short-term effect on the upper airway, with significant short-term increases in the nasal cavity and nasopharyngeal volume and statistically insignificant increases in oropharyngeal and hypopharyngeal volumes.

### 4.2. Agreements and Disagreements

In contrast to other reviews regarding changes in the airway after treatment with RME, a systematic review and meta-analysis conducted by other researchers showed statistically significant increases in both nasopharyngeal and oropharyngeal volumes [35]. This is contrary to the results of our study, which only showed a significant increase in the nasal cavity and nasopharyngeal volume, which may be due to the fact that the patients included in the present study were limited to the use of the Hyrax arch dilator, whereas the review mentioned above was categorized on the basis of patients with malocclusion. At the same time, another study reached similar conclusions in terms of dilation outcomes [36]. Liu et al. noted that nasal cavity width and nasal floor width were increased significantly while no significant expansion of other airway volumes was observed during MARPE. The present study was analyzed on the basis of the results of the retention period recorded in the included literature, which varied from 3 to 12 months, whereas in another retrospective study, the retention period was fixed at three months, and similar conclusions were drawn as in the present literature, which suggests that dilatation of the patient’s airway may still occur during the retention period, and the length of the retention period has an effect on the changes in the airway.

### 4.3. Innovations and Limitations

This review is the first to specifically summarize the results of the impact of Hyrax dilator application through CBCT 3D analysis measurements of the upper airway and would be important for further research efforts. We use the Hyrax dilator for the enlarged upper airway of the RME, aiming for long-term improvement in sleep apnea syndrome and maxillary transverse hypoplasia, as well as protection and maintenance of the enlarged upper airway outcomes. In addition, this review provides specific definitions of quantitative data for all included studies, including specific definitions of the Hyrax expander, specific definitions of measures, and a qualitative evaluation of the results of the studies, as well as the literature itself, to ensure the accuracy of comparisons. Specialized research on this type of expander will lead to more refinement of current and subsequent expansion treatments, and the strengths and weaknesses of the various expanders will be further demonstrated. The use of CBCT measurements as the primary measurements will make the results of the study more scientific and objective.

In terms of limitations, this review lacked differences in upper airway changes in the mid- and long-term follow-up after Hyrax treatment, which has been shown to exist in RME in previous literature [17], and this analysis failed to eliminate these differences. In addition, the quality of the evidence included in the studies was generally low, particularly the lack of study blinding, which resulted in the randomization of the studies not being guaranteed, and the lack of healthy control, which did not allow for the elimination of the relationship between the patient’s airway changes and their growth, leading to questions about the extent to which the dilator was effective.

## 5. Conclusions

After performing this systematic review and meta-analysis, the researchers concluded that RME with Hyrax expander showed significant increases in the nasopharynx and statistically insignificant increases in the nasal cavity, oropharynx, and hypopharynx. Despite that Hyrax expander patients’ upper airway was able to provide a significant short-term dilation, we have not been able to determine that this expander significantly dilates the patient’s upper airway due to the clinical studies used in this meta-analysis has high risks of bias and low quality of scientific evidence. The available literature consistently supports the dilating effect of RME on the upper airway. However, scientific evidence should be interpreted and considered with caution due to the small number of results, the limitation of the effect on short-term data, significant differences in measurements, and the lack of basic methodological standards. In addition, future studies should address issues such as the long-term results of Hyrax arch dilation and RME.

## Figures and Tables

**Table 1:** Electronic databases used and search strategies.

Database	Search Strategy	Retrieved Data
PubMed	(“rapid maxillary expansion” [MeSH Terms] OR (“rapid” [All Fields] AND “maxillary” [All Fields] AND “expansion” [All Fields]) OR “rapid maxillary expansion” [All Fields] OR “maxillary expansion” [All Fields]) AND (“upper airway” [MeSH Terms] OR (“upper” [All Fields] AND “airway” [All Fields]) OR “upper airway” [All Fields])	79
Embase	‘rapid maxillary expansion’/exp OR ‘rapid maxillary expansion’ OR (‘maxillary expansion’/exp OR ‘maxillary expansion’) AND (‘upper airway’/exp OR ‘upper airway’)	133
Web of Science	(rapid maxillary expansion (Topic) OR maxillary expansion (Topic)) AND upper airway (Topic)	208
Cochrane Library	(rapid maxillary expansion): ti,ab,kw OR (maxillary expansion): ti,ab,kw AND (“upper airway”): ti,ab,kw	410

**Table 2:** Characteristics of included studies.

Study	Country	Study Design	Retention
Abdalla et al., 2019 [15]	Denmark Australia	Retrospective study	6 months
Aljawad et al., 2021 [16]	Korea	Retrospective study	4 months
Badreddine et al., 2017 [17]	Brazil	Retrospective study	3 months
Caruso et al., 2023 [18]	Italy	Retrospective study	12 months
Chang et al., 2017 [19]	China	Randomized controlled trial	1 month
Chang et al., 2013 [20]	USA	Prospective study	3–4 months
Cheung et al., 2021 [21]	Australia	Randomized controlled trial	6 months
Kavand et al., 2019 [22]	USA	Retrospective study	6 months
Kecik et al., 2017 [23]	Turkey	Retrospective study	until a 2 mm overjet was received
Korayem et al., 2023 [24]	Saudi Arabia	Retrospective study	6 months
Lanteri et al., 2020 [25]	Italy	Retrospective study	10 months
Mordente et al., 2016 [26]	Portuguese	Retrospective study	3 months
Pangrazio-Kulbersh et al., 2012 [27]	USA	Prospective study	6 months
Smith et al., 2012 [28]	USA/Egypt	Prospective study	Until the palatal cusps of the maxillary first molars contacted the buccal cusps of the mandibular first molars
Tsolakis et al., 2023 [29]	Greece	Prospective study	6 months
Zeng et al., 2013 [30]	China	Prospective study	3 months
**Study**	**N of Treatment Group (Male/Female)**	**Age of Treatment Group (x ± s)**	**Control Group (in the Same Period)**
Abdalla et al., 2019 [15]	26 (not clear)	12.3 ± 2.3	YES
Aljawad et al., 2021 [16]	17 (not clear)	12.6 ± not clear	YES
Badreddine et al., 2017 [17]	39 (23/16)	9.7 ± 2.28	YES
Caruso et al., 2023 [18]	14 (6/8)	8 ± 1	NO
Chang et al., 2017 [19]	18 (7/11)	9.8 ± 1.3	NO
Chang et al., 2013 [20]	14 (5/9)	12.9 ± not clear	NO
Cheung et al., 2021 [21]	19 (not clear)	13.8 ± not clear	YES
Kavand et al., 2019 [22]	Tooth-borne: 18 (8/10) Bone-borne: 18 (6/12)	Tooth-borne:14.4 ± 1.3 Bone-borne: 14.7 ± 1.4	NO
Kecik et al., 2017 [23]	UCLP patients: 23 (12/11) Non-cleft Class III subjects with maxillary retrusion patients: 26 (14/12)	UCLP patients: 8.3 ± 2.4 Non-cleft Class III subjects with maxillary retrusion patients: 8.1 ± 2.5	NO
Korayem et al., 2023 [24]	52 (not clear)	Not clear	YES
Lanteri et al., 2020 [25]	22 (not clear)	8.1 ± not clear	YES
Mordente et al., 2016 [26]	10 (not clear)	11.1 ± not clear	YES
Pangrazio-Kulbersh et al., 2012 [27]	Banded: 13 (7/6) Bonded: 10 (5/5)	Banded: 12.6 ± 1.8 Bonded: 13.5 ± 2.1	NO
Smith et al., 2012 [28]	20 (8/12)	12.3 ± not clear	NO
Tsolakis et al., 2023 [29]	14 (not clear)	11.12 ± not clear	NO
Zeng et al., 2013 [30]	16 (10/6)	12.73 ±1.7	NO
**Study**	**Methods of Control Group**	**N of Control Group (Male/Female)**	**Age of Control Group (x ± s)**
Abdalla et al., 2019 [15]	Orthodontic treatment without RME	26 (12/14)	12.33 ± not clear
Aljawad et al., 2021 [16]	Orthodontic treatment without RME	17 (not clear)	12.3 ± not clear
Badreddine et al., 2017 [17]	Duly treated using the same standard procedure employed in the treatment group	16 (not clear)	8.8 ± not clear
Caruso et al., 2023 [18]	NO		
Chang et al., 2017 [19]	NO		
Chang et al., 2013 [20]	NO		
Cheung et al., 2021 [21]	Hybrid-Hyrax expander or Keles keyless expander	Hybrid-Hyrax:19 (not clear) Keles:13 (not clear)	Hybrid-Hyrax:14.3 ± not clear Keles:14.6 ± not clear
Kavand et al., 2019 [22]	NO		
Kecik et al., 2017 [23]	NO		
Korayem et al., 2023 [24]	Fixed orthodontic appliances for minor malocclusions without extractions (without RME)	52 (not clear)	Not clear
Lanteri et al., 2020 [25]	Slow maxillary expansion (SME)	22 (not clear)	8.2 ± not clear
Mordente et al., 2016 [26]	Fan-Type, iMini-M, or iMini-B	10 (not clear) 10 (not clear) 10 (not clear)	10.5 ± not clear 10.5 ± not clear 12.5 ± not clear
Pangrazio-Kulbersh et al., 2012 [27]	NO		
Smith et al., 2012 [28]	NO		
Tsolakis et al., 2023 [29]	NO		
Zeng et al., 2013 [30]	NO		
**Study**	**Ethical Approval**	**Eligibility Criteria**	**Measurements**
Abdalla et al., 2019 [15]	YES (only from Denmark)	Inclusion criteria: YES Exclusion criteria: YES	3D imaging and analysis software for clear labeling.
Aljawad et al., 2021 [16]	YES	Inclusion criteria: YES Exclusion criteria: N/A	3D imaging and analysis software for clear labeling.
Badreddine et al., 2017 [17]	YES	Inclusion criteria: N/A Exclusion criteria: YES	3D imaging and analysis software for clear labeling.
Caruso et al., 2023 [18]	YES	Inclusion criteria: YES Exclusion criteria: YES	N/A
Chang et al., 2017 [19]	YES	Inclusion criteria: YES Exclusion criteria: YES	3D imaging and analysis software for clear labeling.
Chang et al., 2013 [20]	YES	Inclusion criteria: YES Exclusion criteria: YES	3D imaging and analysis software for clear labeling.
Cheung et al., 2021 [21]	YES	Inclusion criteria: YES Exclusion criteria: YES	3D imaging and analysis software for clear labeling.
Kavand et al., 2019 [22]	YES	Inclusion criteria: YES Exclusion criteria: N/A	3D imaging and analysis software for clear labeling.
Kecik et al., 2017 [23]	N/A	Inclusion criteria: YES Exclusion criteria: YES	Analysis software for clear labeling but 3D imaging not for clear labeling.
Korayem et al., 2023 [24]	YES	Inclusion criteria: YES Exclusion criteria: N/A	Analysis software for clear labeling but 3D imaging not for clear labeling.
Lanteri et al., 2020 [25]	YES	Inclusion criteria: YES Exclusion criteria: YES	3D imaging and analysis software for clear labeling.
Mordente et al., 2016 [26]	N/A	Inclusion criteria: YES Exclusion criteria: N/A	3D imaging and analysis software for clear labeling.
Pangrazio-Kulbersh et al., 2012 [27]	YES	Inclusion criteria: YES Exclusion criteria: YES	Analysis software for clear labeling but 3D imaging not for clear labeling.
Smith et al., 2012 [28]	YES	Inclusion criteria: YES Exclusion criteria: N/A	3D imaging and analysis software for clear labeling.
Tsolakis et al., 2023 [29]	YES	Inclusion criteria: YES Exclusion criteria: YES	3D imaging and analysis software for clear labeling.
Zeng et al., 2013 [30]	YES	Inclusion criteria: YES Exclusion criteria: YES	3D imaging and analysis software for clear labeling.

**Table 3:** Main findings of included studies.

Study	Results	Nasal Cavity Border	Conclusion
Abdalla et al., 2019 [15]	Upper airway space dimensions (total and MCA)	N/A	No significant effect occurred in upper airway volume or MCA in children.
Aljawad et al., 2021 [16]	Upper airway space dimensions (nasopharyngeal, retropalatal and retroglossal airway)	N/A	An increase occurred in upper airway dimensions.
Badreddine et al., 2017 [17]	Nasal cavity width	N/A	Significant increases occurred in all the skeletal and soft tissue variables (*p* ≤ 0.05).
Caruso et al., 2023 [18]	Skeletal variables; dental variables; upper airway space dimensions	N/A	A significant increase occurred in the upper airway linear measurements and the nasopharyngeal and oropharyngeal dimensions (*p* ≤ 0.05).
Chang et al., 2017 [19]	Upper airway space dimensions (nasal cavity, nasopharyngeal, oropharyngeal, hypopharyngeal)	N/A	An increase occurred in the volume of nasal and nasopharynx cavities, but no significant effect occurred on oropharynx and hypopharynx cavities.
Chang et al., 2013 [20]	Upper airway space dimensions (retropalatal, retroglossal, total)	Labeled clearly	A moderate increase occurred in the cross-sectional area of the upper airway.
Cheung et al., 2021 [21]	Upper airway space dimensions (nasal cavity, nasopharyngeal, and oropharyngeal)	Labeled clearly	A relatively small increases occurred in total upper airway volume and its separate compartments.
Kavand et al., 2019 [22]	Upper airway space dimensions (nasal cavity, nasopharyngeal, and oropharyngeal)	Labeled clearly	A significant increase occurred in nasal cavity and nasopharynx volume (*p* ≤ 0.05).
Kecik et al., 2017 [23]	Lateral cephalometric radio graphs of upper airway (Ad1-PNS, Ad2-PNS, nasopharynx, oropharynx, Soft palate)	N/A	A significant increase occurred in the nasopharyngeal volume measurements (*p* < 0.001).
Korayem et al., 2023 [24]	Upper airway space dimensions (total and MCA)	N/A	No significant effect occurred in upper airway volume or MCA in children.
Lanteri et al., 2020 [25]	Upper airway space dimensions (nasal cavity (NCavV), nasopharyneal (NsPxV), and left and right maxillary sinuses (MSV))	Labeled clearly	Significant increases occurred in nasal cavity and nasopharynx after treatment with both appliances (*p* ≤ 0.05).
Mordente et al., 2016 [26]	Upper airway space dimensions (nasal cavity, and oropharyngeal)	Not mentioned	An increase occurred in the nasal passage volume. No significant effect occurred in oropharyngeal volume.
Pangrazio-Kulbersh et al., 2012 [27]	Linear, angular, and volumetric measurements of the upper airway	N/A	An increase occurred in upper airway dimensions, including palatal suture and demonstrated anterior and posterior skeletal widening of the nasal cavity, with corresponding soft tissue changes and total airway volume.
Smith et al., 2012 [28]	Upper airway space dimensions (nasal cavity, nasopharyngeal, oropharyngeal, hypopharyngeal)	Labeled clearly	Significant increases occurred in nasal cavity volume, nasopharynx volume, anterior and posterior facial heights, and palatal and mandibular planes (*p* ≤ 0.05).
Tsolakis et al., 2023 [29]	Upper airway space dimensions (total and MCA)	N/A	Significant increases occurred the airway volume and minimal cross-sectional area in the nasal passage.
Zeng et al., 2013 [30]	Upper airway space dimensions (lower nasal cavity, nasopharyngeal, oropharyngeal).	Labeled clearly	An increase occurred in upper airway dimensions, including the nasal cavity and the pharyngeal volumes, but the influence on the pharyngeal airway is limited.

**Table 4:** Risk of bias of included RCT studies.

Study	Adequate Sequence Generation	Allocation Concealment	Incomplete Outcome Data	Selective Reporting	Blinding of Outcome Assessment	Other Bias
Chang et al., 2017 [19]	Low	Low	Low	Low	Low	Moderate
Cheung GC et al., 2021 [21]	Low	Low	Low	Low	Low	Low

**Table 5:** Risk of bias of included non-RCT studies.

Study (b)	[SD]	[R]	[SS]	[SC]	[C]	[FU]	[OB]	[DO]	[IV]	[A]	[B]
Abdalla et al., 2019 [15]	0	0	1	1	1	0	1	0	1	1	0
Aljawad H et al., 2021 [16]	0	0	0	0	0	0	1	0	1	1	0
Badreddine et al., 2017 [17]	0	0	1	0	1	1	1	0	1	1	0
Caruso S et al., 2023 [18]	0	0	0	0	0	0	1	0	1	1	0
Chang DT et al., 2017 [19]	0	0	0	0	0	0	1	0	0	0	0
Chang Y et al., 2013 [20]	0	0	0	0	0	0	1	0	1	1	0
Cheung GC et al., 2021 [21]	0	0	1	0	0	0	1	0	0	0	0
Kavand et al., 2019 [22]	0	0	0	1	0	1	1	0	0	1	0
Kecik D et al., 2017 [23]	0	0	0	0	0	0	1	0	1	1	0
Korayem MA et al., 2023 [24]	0	0	1	0	0	0	1	0	1	1	0
Lanteri V et al., 2020 [25]	0	0	1	0	0	0	1	0	1	1	0
Mordente CM et al., 2016 [26]	0	0	1	0	0	0	1	0	1	1	0
Pangrazio-Kulbersh et al., 2012 [27]	1	1	1	0	0	0	1	0	0	1	0
Smith et al., 2012 [28]	0	0	1	1	0	0	1	0	1	1	0
Tsolakis IA et al., 2023 [29]	1	1	1	0	0	0	1	0	1	1	0
Zeng et al., 2013 [30]	1	0	0	1	0	0	1	0	1	0	0
Total	3	2	9	4	2	2	16	0	12	13	0

Note: Abbreviations: A, Analysis; B, Blinding; C, Control; DO, Dropouts; FU, Follow-up; IV, Intervention; OB, Objective; R, Randomization; SC, Selection criteria; SD, Study design; SS, Sample size.

**Table 6:** Characteristics of evidence quality classification.

Indicators of Evaluation	Number of Articles	Assessment of Evidence Quality					
		Risk of Bias		Inconsistency	Indirectness	Inaccuracy	Publication Bias
Nasal cavity	8	High risk		Low risk	Low risk	Low risk	Low risk
Nasopharynx	8	High risk		High risk	Low risk	Low risk	Low risk
Oropharynx	6	High risk		Low risk	Low risk	High risk	Low risk
Hypopharynx	3	High risk		Low risk	Low risk	High risk	Low risk
**Number of Patients**	**Effect Size**				**Quality of Evidence**	**Degree of Importance**	
	**MD**		**95% CI**				
358	1.92		[1.06–2.79]		Moderate	Important	
288	0.69		[0.09–1.28]		Low	Important	
202	0.03		[−1.16–1.22]		Low	Not important	
114	0.05		[−0.30–0.41]		Low	Not important	

## Data Availability

The data presented in this study are available on request from the corresponding authors.
